# Tobacco, Alcohol, and Processed Food Industries – Why Do Public Health Practitioners View Them So Differently?

**DOI:** 10.3389/fpubh.2016.00064

**Published:** 2016-04-11

**Authors:** Katherine Smith, Lori Dorfman, Nicholas Freudenberg, Benjamin Hawkins, Shona Hilton, Oliver Razum, Heide Weishaar

**Affiliations:** ^1^Global Public Health Unit, University of Edinburgh, Edinburgh, UK; ^2^Berkeley Media Studies Group, Public Health Institute, and School of Public Health, University of California Berkeley, Berkeley, CA, USA; ^3^City University New York School of Public Health, New York, NY, USA; ^4^London School of Hygiene and Tropical Medicine, London, UK; ^5^MRC/CSO Social and Public Health Sciences Unit, University of Glasgow, Glasgow, UK; ^6^School of Public Health, Bielefeld University, Bielefeld, Germany

**Keywords:** tobacco, alcohol, processed food, obesity, prevention, health policy, non-communicable diseases

The epidemiological transition, the shift from infectious to chronic non-communicable diseases (NCDs), is well advanced in most European countries. Viewed from one perspective, we can understand this by focusing on changes to people’s lifestyles and behaviors. However, a contrasting view draws attention to broader, social and environmental features that are unfavorable to health. The World Health Organization (WHO) slogan “Make the healthy choice the easier choice” attempts to bridge these two views. It not only recognizes the choices that individuals have in consuming healthier products or taking exercise but also seems to acknowledge that consumer “choices” are heavily influenced by other factors beyond the individual’s power. Among these factors are the strategies that corporations pursue to make unhealthy choices more likely. Thus, public health is inevitably confronted with the question of how to interact with commercial interests when it comes to tackling the NCD epidemic.

The engagement of public health practitioners and researchers with tobacco industry is now highly controversial leading many scholars to eschew interactions with the industry (1). Reflecting this, many leading journals now refuse to publish tobacco industry funded research (2). Yet, many public health officials consider dealing with the processed food, soft drink, and alcohol industries as normal practice, treating them as legitimate partners in improving population health (3). What is the rationale behind such different approaches to dealing with these industries, given that their products have a significant adverse impact on population health and their business strategies use similar approaches when it comes to marketing, product design, policy influence, and challenging evidence of harm (4, 5)?

In part, the answer lies in the extensive research linking the consumption of tobacco products to a range of negative health outcomes and the activities of the tobacco industry (4). As a result of a series of internal leaks followed by litigation against major tobacco companies in the US, internal tobacco company documents have entered the public realm (6). These highlight that senior managers of tobacco companies have (among other things): lied about how addictive tobacco products are, worked to increase the addictiveness of products; targeted young children as new product “markets”; and worked to restrain policies that aim to limit tobacco consumption and the influence of tobacco companies [see, e.g., Ref. (7)]. These revelations led to the conceptualization of the tobacco industry as a key NCD “vector” (4). Public health efforts to denormalize tobacco have effectively undermined public and political legitimacy of tobacco industry actors in selected policy contexts, where industry representatives are increasingly excluded from tobacco control debates (8). However, the growing popularity of e-cigarettes is threatening the cohesion of the movement to control tobacco and offering new avenues through which tobacco industry actors can access policy makers (9).

Processed food, alcohol, and soft drink industries tend to argue (as the tobacco industry used to claim) that it is the individual’s personal responsibility to choose healthier options, for example, by exercising, eating healthy diets, and reducing the intake of less healthy products. The CEO of PepsiCo, Indra Nooyi, has, for example, argued that PepsiCo is an “ethical” company. She pointed out that her company offers a selection of products, ranging from healthy to less healthy. This view is frequently reinforced through advertising, news stories and television programs and, in many cases, government policies. From this perspective, considering the corporate interests relating to the production and marketing of products as social determinants of NCDs makes sense. However, these actors often portray themselves as “part of the solution” (10) to the health crises that their products exacerbate. Thus, the development of reformulated products, changes to labeling, support for “educational” initiatives, and the implementation of self-regulatory codes of practice are framed as appropriate strategies toward the control of NCDs.

The health harms associated with the tobacco, alcohol, and processed food industries are significant. Research shows that alcohol and obesity contribute significantly to unfavorable health outcomes (e.g., in pregnancy), often in a magnitude comparable to that of tobacco (4, 11). Evidence also suggests that the health-related costs of products across these industries are similar and are perhaps highest for obesity (4, 12), rather than for tobacco. It is also clear that commercial interests and strategies across these sectors are similar, with industry representatives actively working to influence public and policy debates with a view to minimize the potential for regulation and maximize profit. Three examples suffice as follows:

- Recent research examining how alcohol industry actors in the UK have attempted to block policy proposals for minimum unit pricing identify strategies for policy influence that have been widely used by the tobacco industry, including efforts to shape the available evidence base (and the public’s, the media’s, and policymakers’ understandings of the available evidence), direct and indirect lobbying, links to more credible organizations such as think tanks, and efforts to shape public perceptions of the industry (13), often *via* the media (14).
- Corporate social responsibility (CSR) strategies are employed across all of these industries as means of shaping political contexts and informing public perceptions and consumption patterns. Despite these efforts to enhance their credibility, however, soda companies behave irresponsibly, for example, by explicitly targeting children and setting goals to increase consumption (15).
- In 2009–2012, a coalition of more than 50 food and beverage companies in the US invested US-$175 million to successfully lobby the Obama Administration not to pursue tougher (albeit still voluntary) nutritional standards for food items marketed to children (5).

Why then, in light of such striking similarities, do people appear to view the tobacco industry so differently to processed food, soft drinks, and alcohol industries? It may be, as Collin (1) argues – partly a consequence of the success of the tobacco control movement in promoting “tobacco exceptionalism” – the idea that the tobacco industry – as a result of both the health costs of tobacco products and our knowledge about prior industry behavior – requires a *uniquely* strict approach to protecting public health policy from the interference of industry.

Although it may be argued that tobacco is a uniquely harmful product – when used precisely as intended by manufacturers, tobacco will kill 50% of long-term users – a growing body of research suggests that the industry which produces it is far from unique as a vector of disease. Given the magnitude of the public health challenge posed by NCDs, we need to move beyond identifying the current, contradictory approaches to these different industries. We propose four priorities for public health research. They should help us to better comprehend how these key industries are perceived, and how they influence the way politics and the public accepts them and their strategies. Researchers must:

1. develop tools to better understand how processed food, soft drinks, and alcohol industries influence public, media, political, and policy debates,
2. examine how policymakers, journalists, and the public view each of these industries and the products they market, and why,
3. consider how research in this area might support policies that are effective and evidence informed, and will contribute toward promoting and protecting the public’s health, and
4. investigate the complex network of actors that constitute each of these distinct industries and identify any interactions between them. In this way, we can elucidate interests, strategies, and actions that are common across industries.

In moving this agenda forward, public health researchers need to make space for developing “charismatic ideas” – convincing alternative scenarios of a healthier future (5). This is necessary to identify possibilities and new avenues to reduce harmful corporate influences on health.

## Author Contributions

KS and OR drafted the text with the input of all authors. All authors revised the work critically for important intellectual content, approved the final version to be published, and agreed to be accountable for all aspects of the work in ensuring that questions related to the accuracy or integrity of any part of the work are appropriately investigated and resolved.

## Conflict of Interest Statement

This opinion piece is based on a workshop held at the 8th European Public Health Conference, 14–17 October 2015, jointly sponsored by Policy and Politics and the University of Glasgow MRC/CSO Social and Public Health Sciences Unit. All authors declare that the research was conducted in the absence of any other commercial or financial relationships that could be construed as a potential conflict of interest.
